# Editorial: Organ support in cardiac intensive care

**DOI:** 10.3389/fcvm.2024.1510197

**Published:** 2024-10-31

**Authors:** Guo-wei Tu, Sascha Treskatsch, Takatoshi Kasai

**Affiliations:** ^1^ Cardiac Intensive Care Center, Zhongshan Hospital, Fudan University, Shanghai, China; ^2^ Department of Anesthesiology and Intensive Care Medicine, Charité—Universitätsmedizin Berlin, Corporate Member of Freie Universität Berlin and Humboldt-Universität Zu Berlin, Campus Benjamin Franklin, Berlin, Germany; ^3^ Department of Cardiovascular Biology and Medicine and Cardiovascular Respiratory Sleep Medicine, Juntendo University Graduate School of Medicine, Tokyo, Japan

**Keywords:** extracorporeal membrane oxygenation, intra-aortic balloon pump, pulmonary embolism, diaphragmatic dysfunction, dynamic arterial elastance

**Editorial on the Research Topic**
Organ support in cardiac intensive care

Severe heart disease can trigger a cascade of systemic organ dysfunctions, encompassing respiratory distress, acute kidney injury and gastrointestinal ischemia. Some patients may even require extracorporeal life support to bridge to recovery, transplantation or decision making (1–3). Consequently, the cardiac intensive care unit (ICU) demands robust organ support capabilities. The prevailing challenges in this domain encompass the maintaining organ functionality in critically ill individuals and the prudent use of extracorporeal life support technologies.

In this special issue, we have invited researchers from around the globe to focus on the issue of organ support in cardiac ICU. We have received a total of 26 submissions, indicating that this field has indeed captured the interest and attention of the academic community. We would like to take this opportunity to express our sincere appreciation to all contributors and readers.

We are pleased to accept 8 papers on the research topic of Organ Support in Cardiac Intensive Care (https://www.frontiersin.org/research-topics/60649/organ-support-in-cardiac-intensive-care), comprising 4 original research articles, 1 review, and 3 case reports. The studies mainly focused on hemodynamic monitoring, extracorporeal membrane oxygenation (ECMO) and Intra-aortic Balloon Pump (IABP) support.

Massive pulmonary embolism (MPE) is one of the leading causes of preventable cardiovascular mortality and morbidity. ECMO may hold a pivotal role in the treatment of MPE (4–6). Davies and Hart have explored the utility of ECMO for MPE. They discussed the pathophysiology of MPE and how ECMO can provide hemodynamic support by oxygenating the blood and right ventricular unloading. The review also scrutinized experimental models, clinical guidelines, and the different strategies for using ECMO in MPE, such as a bridge to intervention or as standalone therapy. Despite the potential benefits, the application of ECMO is not without risks, including systemic inflammation and hemorrhagic complications.

IABP is sometimes coupled with Venoarterial extracorporeal membrane oxygenation (VA-ECMO) to treat patients with cardiogenic shock. Wang et al. attempted to evaluate the impact of the IABP strategy on survival rates and the incidence of vascular complications in adult patients with cardiogenic shock who are undergoing VA-ECMO therapy. This systematic review analyzed the effectiveness of VA-ECMO treatment, with and without the addition of IABP, in adult patients experiencing cardiogenic shock. The study reavled that the concomitant use of IABP with VA-ECMO was associated with a decrease in-hospital mortality. However, no statistically significant differences were observed between the two treatment groups, including rates of neurological, gastrointestinal, limb-related, bleeding, and infectious complications.

The evaluation of left ventricular (LV) function is crucial in management of patients with VA-ECMO, however, the most widely used measures of LV performance are substantially load-dependent (7). Lakatos et al. conducted a multicenter observational study named MIX-ECMO, which aimed to assess the LV contractility in patients undergoing VA-ECMO using a non-invasive myocardial work index. The study sought to determine the prognostic value of this index in predicting the success of weaning from VA-ECMO support and explored its potential advantages over conventional parameters of LV function.

Dynamic arterial elastance (Eadyn) has emerged as a parameter of interest for predicting the hemodynamic response to various interventions (8). In the systematic review and meta-analysis by Zhou et al. the authors investigated the predictive performance of Eadyn for mean arterial pressure (MAP) response to norepinephrine weaning in mechanically ventilated patients suffering from vasoplegic syndrome. In this study, Eadyn was found to be a reliable predictor of MAP response, with a high area under the hierarchical summary receiver operating characteristic curve (AUHSROC). This indicated its potential use in guiding clinical decisions regarding norepinephrine weaning.

Huai et al. investigated diaphragmatic dysfunction in patients following cardiac surgery, aiming to assess its impact on prognosis and identify potential risk factors. The study revealed a 40.7% incidence of early postoperative diaphragmatic dysfunction, which might be associated with a higher likelihood of requiring noninvasive ventilation and increased oxygen support. Although the difference was not statistically significant, there was a trend towards longer ICU and postoperative hospital stays for those with diaphragmatic dysfunction. These findings are crucial for guiding respiratory care and rehabilitation approaches during the postoperative phase of cardiac surgery (9).

In the research collection of Organ Support in Cardiac Intensive Care, three interesting case reports were included. Yang et al. presented a 42-year-old male who developed multiple organ embolism and intestinal necrosis subsequent to IABP use following percutaneous coronary intervention. The case report emphasized the risk of severe complications associated with IABP use, highlighting the importance of vigilant post-operative care and the need for prompt diagnosis and intervention to address such complications. Jin et al. reported the challenges in managing an eight-year-old female with restrictive cardiomyopathy (RCM), a rare heart condition often leading to severe heart failure. The patient was treated with ECMO after experiencing cardiac arrest, but later succumbed to heart failure due to persistent atrial flutter. The study identified a novel TNNI3 variant linked to the patient's RCM and discussed the molecular impact of this genetic mutation on cardiac function. Jiang et al. introduced an innovative approach to managing insufficient left ventricular unloading in two patients on VA-ECMO. In both cases, the application of gentle chest compressions created an active drainage mechanism for the LV, preventing the formation of intracardiac thrombi and improving pulmonary circulation. The report highlighted the safety and efficacy of this method, as no life-threatening complications or technical issues were encountered. Both patients recovered well, with follow-up examinations showing full capacity for independent living and no residual organ dysfunction. The study concluded that gentle chest compressions could be a viable treatment for insufficient LV unloading in VA-ECMO patients, offering a less invasive and potentially safer alternative to mechanical assist devices.

This research collection is dedicated to the critical issue of organ support in critically ill cardiac patients. We extend our heartfelt thanks to the authors, reviewers, and editor team for their indispensable efforts and dedication. We hope the insights gained from this collection will not only bring everyone up to speed on the latest advancements in the field but also serve as a valuable reference for upcoming clinical and research endeavors.
